# Impact of Coronavirus Disease 2019 on Unresectable Hepatocellular Carcinoma Treated with Atezolizumab/Bevacizumab

**DOI:** 10.3390/jcm13051335

**Published:** 2024-02-27

**Authors:** Yun Beom Sang, Chaeryoung Lee, Seul-Gi Kim, Boyoung Lee, Beodeul Kang, Chan Kim, Hong Jae Chon

**Affiliations:** 1 Medical Oncology, Department of Internal Medicine, CHA Bundang Medical Center, CHA University, Seongnam 13497, Republic of Korea; ybsang85@chamc.co.kr (Y.B.S.); sophia311@chamc.co.kr (S.-G.K.); wb0707@cha.ac.kr (B.K.); 2 Division of Infectious Diseases, Department of Internal Medicine, CHA Bundang Medical Center, CHA University, Seongnam 13497, Republic of Korea; folareta@chamc.co.kr; 3 Division of Allergy and Respiratory Diseases, Department of Internal Medicine, Soonchunhyang University Hospital, Seoul 04401, Republic of Korea; etboss2@schmc.ac.kr

**Keywords:** coronavirus disease 2019, unresectable hepatocellular carcinoma, liver function, atezolizumab, bevacizumab

## Abstract

**(1) Background:** The coronavirus disease 2019 (COVID-19) pandemic has proven challenging to the management of patients with cancer, particularly those receiving systemic therapy. This study aimed to evaluate the impact of COVID-19 on patients with unresectable hepatocellular carcinoma (HCC) treated with atezolizumab/bevacizumab. **(2) Methods:** Patients with unresectable HCC who started atezolizumab/bevacizumab treatment between June 2020 and December 2021 at a tertiary cancer center in Korea were included (*n* = 241) and classified according to their COVID-19 status and severity. **(3) Results:** Thirty-five (14.5%) patients with unresectable HCC were diagnosed with COVID-19 during atezolizumab/bevacizumab treatment; 26 (74.2%) and nine (25.7%) in the low- and high-severity groups, respectively. The high-severity group showed higher neutrophil-to-lymphocyte ratios and lactate dehydrogenase levels. Liver and kidney injuries were observed in 31.4% and 17.1% of total patients, respectively. Liver injury was more prominent in patients with pre-existing liver dysfunction at baseline, who were more prevalent in the high-severity group. Atezolizumab/bevacizumab treatment was delayed by a median of 0 (range, 0–21) day in the low-severity group and 12 (range, 0–35) days in the high-severity group. The high-severity group showed worse post-infection progression-free survival (1.1 vs. 4.8 months, *p* = 0.017) and overall survival (2.2 months vs. not reached, *p* = 0.004). **(4) Conclusions:** Patients with impaired liver function at baseline are more susceptible to high-severity COVID-19, which affects atezolizumab/bevacizumab treatment outcomes.

## 1. Introduction

Atezolizumab, an anti-programmed death-ligand 1 antibody, and bevacizumab, an anti-vascular endothelial growth factor antibody, were approved by the Food and Drug Administration (FDA) on May 2020 as a combination immunotherapy for patients with unresectable hepatocellular carcinoma (HCC) [1]. This was the first immunotherapy regimen approved by the FDA for the first-line treatment of unresectable HCC, opening an immunotherapeutic era for HCC [2,3,4,5].

Meanwhile, the global coronavirus disease 2019 (COVID-19) pandemic, caused by severe acute respiratory syndrome coronavirus 2, began around the same period [6]. Although COVID-19 primarily affects the respiratory system, leading to lung injury, such as pneumonia or respiratory failure, there have been many reports indicating that COVID-19 can affect other organs, including the heart, kidneys, liver, and brain [7,8,9,10]. Notably, liver injury, characterized by elevated transaminase levels and/or hyperbilirubinemia, is observed in 15–54% of patients with confirmed COVID-19 [11,12,13,14,15]. Notably, a proportion of patients can be more susceptible to the effects of COVID-19, which could potentially exacerbate existing hepatitis virus infections and cirrhosis, conditions commonly found in most patients with HCC [16,17,18,19]. In addition, patients with cancer who have recently undergone cancer treatment have a higher risk of infection and unfavorable outcomes [20]. However, no study has reported the impact of COVID-19 on patients with HCC undergoing immunotherapy. We aimed to elucidate the impact of COVID-19 on patients with unresectable HCC during first-line atezolizumab and bevacizumab therapy (Ate/Bev).

## 2. Materials and Methods

### 2.1. Ethical Statement

This study was conducted in accordance with the ethical guidelines of the Declaration of Helsinki and approved by the institutional review board of CHA Bundang Medical Center (protocol code CHA-2022-10-062 and date of approval 23 November 2022). Written informed consent was obtained from all participants.

### 2.2. Study Design and Patients

**Figure 1:**
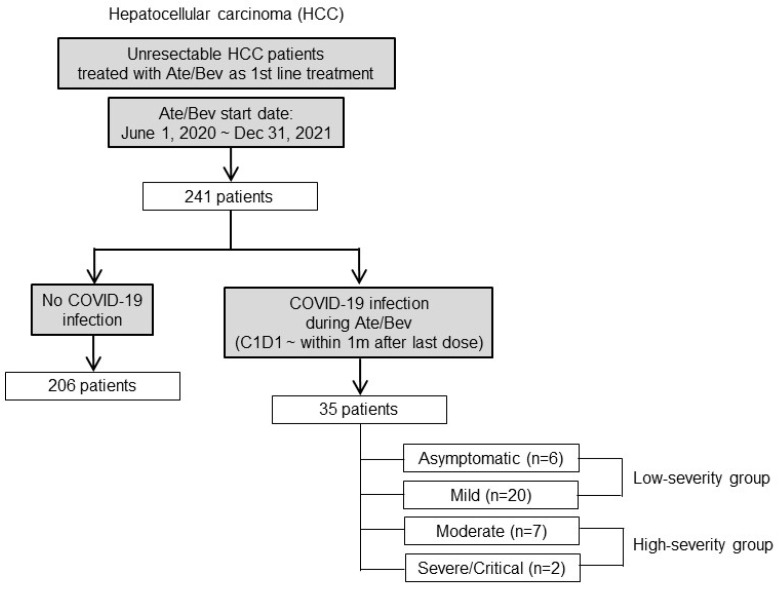
Consort diagram. COVID-19, coronavirus disease 2019; Ate/Bev, atezolizumab/bevacizumab; C1D1, cycle 1 day 1.

Using an observation study design, we enrolled 241 adult patients with unresectable HCC who had started Ate/Bev as first-line systemic therapy between 1 June 2020 and 31 December 2021 at CHA Bundang Medical Center, Korea. Of the 241 patients, 35 were confirmed to be positive for severe acute respiratory syndrome coronavirus 2 (SARS-CoV-2) using reverse transcription polymerase chain reaction-based tests with nasopharyngeal swab samples during Ate/Bev treatment. The patients were classified as asymptomatic, mild, moderate, severe, or critical, according to the Centers for Disease Control and Prevention (CDC) criteria [21]. Patients with asymptomatic or mild infections were categorized into the low-severity group, whereas those with moderate, severe, or critical infections were categorized into the high-severity group (Figure 1). Clinical and laboratory data were obtained from the medical records of the CHA Bundang Medical Center. This study followed the guidelines of the STROBE statement [22].

### 2.3. Treatments and Outcome Evaluation

The patients were treated with atezolizumab (1200 mg fixed dose) and bevacizumab (15 mg/kg) every three weeks as first-line systemic therapy. Dose interruptions or reductions were determined using the IMbrave150 protocol [1]. Ate/Bev treatment was continued until intolerable toxicities and progressive diseases were observed. Response was evaluated every 6 or 9 weeks using computed tomography or magnetic resonance imaging, according to Response Evaluation Criteria in Solid Tumors version 1.1. Overall survival (OS) was defined as the time from treatment initiation to death. Progression-free survival (PFS) was defined as the time between treatment initiation and the date of progressive disease (PD) or death. Post-infection OS (piOS) was defined as the time from COVID-19 onset to the date of death. Post-infection PFS (piPFS) was defined as the time between COVID-19 onset and the date of PD or death. Adverse events (AEs) were assessed using the National Cancer Institute Common Terminology Criteria for Adverse Events version 5.0 [23]. Kidney injury was defined according to the Kidney Disease: Improving Global Outcomes criteria for acute kidney injury (AKI) [24]. Liver injury was defined as an increase in the Child–Pugh (CP) score or liver index.

### 2.4. Statistical Analysis

Patient demographics and clinical characteristics are summarized by frequency and proportion. Independent sample *t*-tests, analysis of variance, and chi-square tests were used to compare the variables. Survival analysis was performed using the Kaplan–Meier method, and subgroups were compared using the log-rank test. Univariate analyses of survival outcomes were performed using the Cox proportional hazards regression analysis. All other statistical analyses were performed using IBM SPSS software (version 18.0). Statistical significance was set at *p* < 0.05.

## 3. Results

### 3.1. Patient Characteristics and COVID-19 Severity

Baseline patient characteristics are presented in Table 1. The median patient age was 62.0 years (interquartile range [IQR]: 55.0–68.0 years), and 86.3% of the patients were male. Most patients had CP class A (75.1%) and Barcelona Clinic Liver Cancer stage C disease (84.6%). Hepatitis B (68.8%) was the most common cause of HCC. Most patients (65.1%) had received at least one prior local therapy for HCC. The median follow-up duration was 10.2 months (IQR: 6.2–14.4 months). The objective response rate of Ate/Bev was 27.8% (95% confidence interval [CI], 22.3–33.9%), and the median PFS and OS were 6.9 (95% CI, 5.0–8.8) and 15.9 (95% CI, 12.3–19.5) months, respectively.

**Figure 2:**
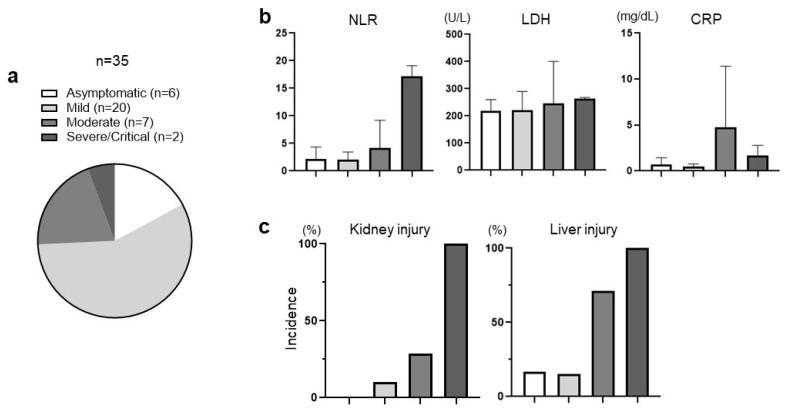
Inflammatory markers and organ dysfunction according to the severity of coronavirus disease 2019. (**a**): The COVID-19 severity of patients according to the CDC severity criteria; (**b**): Inflammatory markers according to COVID-19 severity; (**c**): Organ injury after COVID-19 infection; NLR, neutrophil-to-lymphocyte ratio; LDH, lactate dehydrogenase; CRP, C-reactive protein.

Of the 241 patients, 35 (14.5%) were confirmed to have COVID-19 during the course of Ate/Bev treatment. The severity of COVID-19 in patients, according to the CDC severity criteria, ranged from asymptomatic (*n* = 6) to mild (*n* = 20), moderate (*n* = 7), and severe or critical (*n* = 2) (Figure 2a). The patient groups were categorized, based on their COVID-19 status and severity, into three cohorts: non-infected (*n* = 206, 85.5%), low-severity (asymptomatic and mild, *n* = 26, 10.8%), and high-severity (moderate, severe, and critical, *n* = 9, 3.7%). There were no statistical differences in the baseline clinical parameters among the three groups or between the low- and high-severity groups, except for the CP score of B. The distribution of other clinical factors was comparable between the low- and high-severity groups, except that there were numerically more patients with an Eastern Cooperative Oncology Group status of 1 or 2 and microvascular invasion in the high-severity group than in the low-severity group, although the difference was not statistically significant.

### 3.2. Inflammatory Markers According to COVID-19 Severity

The inflammatory markers in patients with COVID-19 were compared between groups. The median neutrophil-to-lymphocyte ratio (NLR) in patients tended to increase with higher COVID-19 severity, with values of 2.2, 2.1, 4.2, and 17.2 for each group, respectively. The median lactate dehydrogenase (LDH, IU/L) level also showed an increasing trend with COVID-19 severity, with values of 218, 221, 247, and 264 IU/L in each group, respectively. However, the median C-reactive protein (mg/dL) level was highest in the moderate group (4.8), followed by the severe or critical (1.7), mild (0.5), and asymptomatic (0.7) groups (Figure 2b). Detailed laboratory data for each patient can be found in Supplementary Table S1.

### 3.3. Organ Dysfunction and Complications after COVID-19

Of the 35 patients with COVID-19, no patients experienced lung injury, such as pneumonia or respiratory failure, that required hospitalization, and no patients experienced cardiac problems due to COVID-19. In contrast, six of the thirty-five (17.1%) patients experienced AKI within 30 days post-infection (DPI). Notably, all patients with severe COVID-19 developed AKI. All patients who developed AKI after COVID-19 recovered within one month without complications. Eleven out of thirty-five (31.4%) patients experienced liver injury within 30 DPI. Intriguingly, the incidence rate of liver injury was proportional to the severity of COVID-19 (asymptomatic 16.7%, mild 15.0%, moderate 71.4%, and severe 100%) (Figure 2c). Table 2 shows the clinical characteristics, liver injury, and complications associated with COVID-19. Patients with poor baseline liver function were more susceptible to liver injury, with base CP scores of A5 (18.2%), A6 (27.3%), or B7 (54.5%). Patients with poor liver function are more susceptible to complications related to tumor progression or impaired liver function, such as tumor rupture, esophageal/gastric varix bleeding, and hepatic encephalopathy, after COVID-19 (Table 2).

### 3.4. Treatment Delay and Treatment Strategy Changes after COVID-19

The median time from the initiation of Ate/Bev treatment to COVID-19 diagnosis was 165 days (range 21–612 days) in patients with COVID-19. Of the 35 patients with COVID-19, 15 (42.9%) experienced a treatment delay, with a median of 0 days (range, 0–21) among those in the low-severity group and 12 days (range, 0–35) among those with severe infection. Four patients discontinued treatment with Ate/Bev because of complications (three of these patients belonged to the high-severity group).

**Figure 3:**
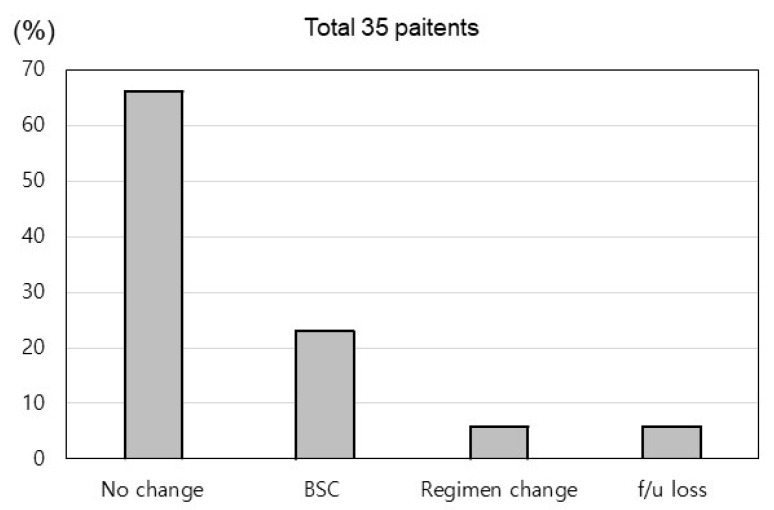
Change in treatment strategy after coronavirus disease 2019. BSC, best supportive care.

Figure 3 illustrates the changes in treatment strategy after COVID-19 in the 35 COVID-19 patients. Most patients (*n* = 23, 65.7%) continued Ate/Bev combination immunotherapy without further complications, except for a slight delay in Ate/Bev treatment. However, eight (22.9%) patients discontinued Ate/Bev treatment and received only best supportive care because of liver function impairment (*n* = 2), deterioration of their general medical condition (*n* = 3), or death (*n* = 3). Three patients passed away due to HCC rupture, gastric perforation, and HEP, respectively, after contracting COVID-19. Two patients (5.7%) had to change their regimen because of PD, and two other patients (5.7%) were lost to follow-up.

### 3.5. Survival Outcomes after COVID-19

**Figure 4:**
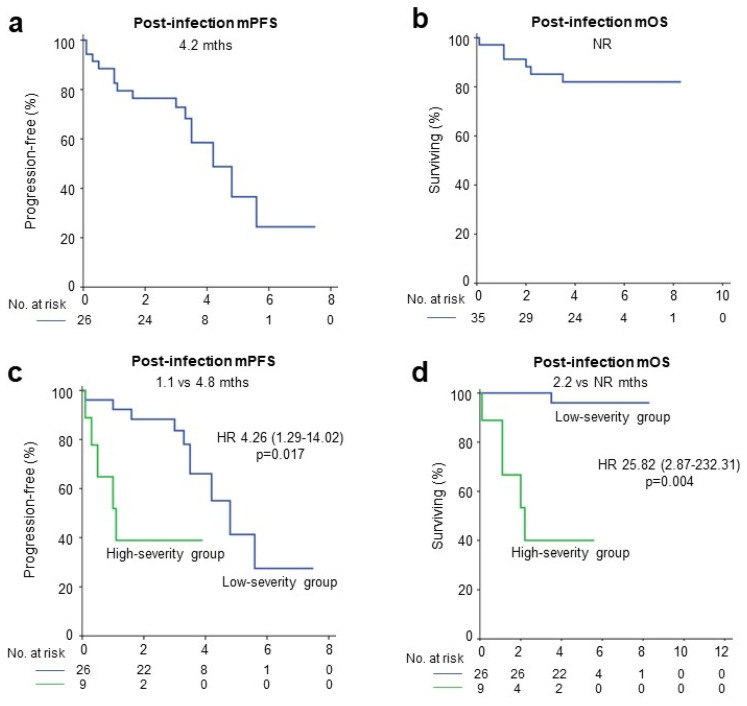
Survival outcomes after coronavirus disease 2019. (**a**): Post-infection median PFS of patients with COVID-19; (**b**): Post-infection median OS of patients with COVID-19; (**c**): Post-infection median PFS according to COVID-19 severity; (**d**) Post-infection median OS according to COVID-19 severity; HR, hazard ratio; NR, not reached.

The median piPFS and piOS of patients with COVID-19 were 4.2 months (95% CI, 2.8–5.6 months) and not reached (NR), respectively (Figure 4a,b). According to COVID-19 severity, the high-severity group showed shorter piPFS and piOS than the low-severity group. The piPFS was 1.1 months in the high-severity group and 4.8 months for the low-severity group (*p* = 0.017). Additionally, the piOS was 2.2 months and NR for the high-severity and the low-severity group, respectively (*p* = 0.004) (Figure 4c,d). As patients receiving Ate/Bev for a longer time are more likely to be exposed to COVID-19, the survival outcomes of patients with COVID-19 were better than those of non-infected patients (Supplementary Figure S1a,b).

## 4. Discussion

Of the 241 patients who started Ate/Bev treatment between 1 June 2020 and 31 December 2021, 35 (14.5%) were diagnosed with COVID-19. Among them, nine (3.7%) were classified in the high-severity group (patients with moderate or severe/critical COVID-19 symptoms) requiring hospitalization, whereas 26 (10.8%) belonged to the low-severity group (patients with asymptomatic or mild COVID-19 symptoms). Patients in the high-severity group demonstrated poorer survival outcomes with shorter piPFS and piOS than those in the low-severity group. Among patients with HCC with concomitant COVID-19, there were no instances of serious respiratory complications, such as acute respiratory distress syndrome; however, the occurrence of liver (31.4%) and kidney injuries (17.1%) was quite noticeable.

Previous studies have shown that the COVID-19 pandemic has significantly affected cancer management. Patients with cancer, particularly those who have recently received cancer treatment, are more vulnerable to infections and tend to have a worse prognosis than the general population [16,20]. This pandemic has led to a decrease in the number of new diagnoses, changes in treatment approaches, and prolonged treatment delays [18]. Concomitant COVID-19 in patients with HCC has also been associated with exacerbated liver conditions, potential hepatic injury, and an increased risk of severe outcomes, including death [16]. However, the impact of COVID-19 on patients with HCC receiving immunotherapy has not been fully elucidated.

There are many known complications associated with COVID-induced liver injury, including inflammatory liver diseases [25], liver fibrosis [26], liver failure [27], and variceal bleeding [28,29]. Among the 35 patients included in our study, hyperbilirubinemia (25.7%), hypoalbuminemia (11.4%), elevated aspartate aminotransferase (20.0%), variceal bleeding (8.6%), elevated alanine aminotransferase (5.7%), and prolonged international normalized ratio (5.7%) were observed. Additionally, patients with poor baseline liver function had higher rates of liver injury. Furthermore, patients with poor liver function were more prevalent in the high-severity group, classified based on COVID-19 symptoms.

Although the majority of patients did not experience significant impacts to their cancer treatment process, except for treatment delays, a fraction of patients had to discontinue Ate/Bev combination therapy and received the best supportive care because of complications related to liver function impairment and deterioration in their general condition. Additionally, three patients died due to complications related to liver injury. Considering that a significant number of patients who develop liver injury also experience liver injury-related complications, we suggest that close attention should be paid to the prevention and treatment of COVID-19 in patients with HCC presenting impaired baseline liver function.

Another important finding was the impact of COVID-19 on survival outcomes. When comparing the survival outcomes of patients with COVID-19 according to COVID-19 severity, patients in the high-severity group exhibited inferior piPFS and piOS compared with those in the low-severity group. It was also noted in our study that the NLR and LDH tended to increase with higher COVID-19 severity. Indeed, inflammatory markers, such as NLR and LDH, are known to help predict the severity and mortality rate in COVID-19 patients [30,31].

There are some limitations to our study. First, this study was conducted retrospectively, relying on patient surveys, external records, and laboratory data obtained during hospital visits. As a result, there may be a decrease in the accuracy of the timing of COVID-19 diagnosis and treatment history. Secondly, although the total number of patients included in this study was substantial, the number of patients with COVID-19 was small, and relatively few patients exhibited typical respiratory symptoms or complications of COVID-19. Due to the small number of patients who received COVID-19 treatment in our study, further trials considering the hepatotoxicity of COVID-19 treatment drugs could provide more clinical insights.

However, we believe that our study effectively demonstrates the detrimental impact of COVID-19 on the prognosis of a significant number of patients with HCC and convincingly highlights the potential disruptions to ongoing treatments. Our findings underscore the importance of vigilant monitoring and follow-up care to optimize treatment outcomes and minimize AEs in patients with HCC during the COVID-19 pandemic.

COVID-19 can induce liver injury and complications in a fraction of patients with unresectable HCC treated with Ate/Bev therapy, leading to treatment delay or discontinuation. Moreover, patients with impaired liver function are more susceptible to severe COVID-19, which affects the clinical outcomes of Ate/Bev treatment. Therefore, close monitoring and appropriate management of these patients will optimize the efficacy of Ate/Bev treatment during the COVID-19 pandemic.

## Figures and Tables

**Table 1:** Baseline characteristics.

Characteristics	All *n* = 241 100.0%	Non-Infected *n* = 206 (85.5%)	* Low-Severity Group *n* = 26 (10.8%)	* High-Severity Group *n* = 9 (3.7%)	* *p*-Value
Age, median (IQR)	62 (55–68)	62 (55–69)	57.5 (54–63)	62 (60–65)	0.398
Male sex	208 (86.3%)	176 (85.4%)	23 (88.5%)	9 (100.0%)	0.287
ECOG status					0.147
0	99 (41.1%)	84 (40.8%)	13 (50.0%)	2 (22.2%)	
1, 2	142 (58.9%)	122 (59.2%)	13 (50.0%)	7 (77.8%)	
Child–Pugh class					0.038
A	181 (75.1%)	155 (75.2%)	21 (80.8%)	4 (44.4%)	
B	60 (24.9%)	51 (24.8%)	5 (19.2%)	5 (55.6%)	
BCLC stage					0.439
B	37 (15.4%)	30 (14.6%)	6 (23.1%)	1 (11.1%)	
C	204 (84.6%)	176 (85.4%)	20 (76.9%)	8 (88.9%)	
Etiology of HCC					
Hepatitis B	162 (67.2%)	137 (66.5%)	19 (73.1%)	6 (66.7%)	0.714
Hepatitis C	17 (7.1%)	15 (7.3%)	1 (3.8%)	1 (11.1%)	0.418
Alcohol	34 (14.1%)	29 (14.1%)	4 (15.4%)	1 (11.1%)	0.752
Other unknown	28 (11.6%)	25 (12.1%)	2 (7.7%)	1 (11.1%)	0.752
MVI	106 (44.0%)	88 (42.7%)	11 (42.3%)	7 (77.8%)	0.067
Extrahepatic spread	149 (61.8%)	132 (64.1%)	13 (50.0%)	4 (44.4%)	0.774

Ate, atezolizumab; Bev, bevacizumab; COVID-i, coronavirus disease infection; ECOG, Eastern Cooperative Oncology Group; BCLC, Barcelona Clinic Liver Cancer; HCC, hepatocellular carcinoma; MVI, microvascular invasion. * Low-severity group; asymptomatic or mild symptoms. * High-severity group; moderate, severe, or critical symptoms. * *p*-Value; between the low-severity group and the high-severity group.

**Table 2:** Clinical characteristics of patients with liver injury or complications after COVID-19.

Age	Sex	Etiology of HCC	BCLC Stage	COVID-19 Severity	* Deterioration of LFT	* CP Score Change	* Event within 60 DPI (Days)	Infection Time from Ate/Bev Start (Days)	Delay of Ate/Bev (Days)
56	M	HBV	C	Asym	-	5	PD (30)	90	0
71	M	Other unknown	B	Asym	-	6	f/u loss (52)	81	0
44	M	HBV	C	Mild	-	5	PD (49)	87	0
61	M	HBV	B	Mild	Bil (1.5→2.4)	5→6	-	285	14
57	M	HBV	C	Mild	Bil (1.1→2.1)	6→7	f/u loss (18)	27	0
41	M	HBV	C	Mild	Bil (2.4→4.3) AST (G0→G1) r-GT (G0→G1)	7→8	ECOG 3 (12)	330	D/C
63	M	Other unknown	C	Mild	Bil (1.4→3.3)	7→9	HEP (56) Death (56)	102	21
46	M	HBV	C	Moderate	Alb (3.5→2.3)	5→6	Esophageal varix bleeding (21)	417	28
51	M	HBV	C	Moderate	Bil (0.7→2.1) Alb (3.2→2.7) AST (G0→G1)	6→8	HCC rupture (30) PD (30) Death (32)	309	14
60	M	HBV	C	Moderate	Bil (1.8→3.9) Alb (2.9→2.4) AST (G2→G3)	6→9	PD (9)	48	9
62	M	HBV	C	Moderate	Bil (1.8→4.7) AST (G1→G2) ALT (G0→G1)	7→9	Gastric varix bleeding (39)	216	D/C
65	M	HBV	C	Moderate	Bil (2.3→4.9) AST (G0→G1)	7→9	ECOG decline (9)	126	D/C
69	F	Other unknown	C	Severe	Alb (3.3→1.3) INR (1.2→2.2) AST (G1→G3) ALT (G0→G3)	7→8	Esophageal varix bleeding (30)	471	D/C
62	M	HCV	C	Critical	Bil (1.3→2.1) INR (2.9→2.4) AST (G1→G3)	7→9	Gastric perforation by PD (3) Death (3)	45	0

BCLC, Barcelona Clinic Liver Cancer; LFT, liver function test; CP, Child–Pugh; DPI, days post-infection; Asym, asymptomatic; Ate, atezolizumab; Bev, bevacizumab; HBV, hepatitis B virus; Bil, bilirubin (mg/dL); INR, international normalized ratio; AST, aspartate aminotransferase (IU/L); Alb, albumin (g/dL); HCC, hepatocellular carcinoma; PD, progressive disease; r-GT, gamma-glutamyl transpeptidase (U/L); D/C, discontinue; BSC, best supportive care; ALT, alanine aminotransferase (IU/L); HCV, hepatitis C virus; f/u, follow-up. * Deterioration of LFT: lab analysis was based on changes that occurred within one month after COVID-19 infection. Changes in grade based on CTCAE 5.0 are indicated. * Significant changes in the CP score within one month after COVID-19 infection, and changes within one month thereafter. * Event within 60 DPI; an event refers to a complication, PD, or death that occurred after COVID-19 infection in the patient.

## Data Availability

Data are contained within the article and Supplementary Materials.

## Supplementary Materials

The following supporting information can be downloaded at: https://www.mdpi.com/article/10.3390/jcm13051335/s1, Figure S1: Survival outcomes according to COVID status; Table S1: Organ dysfunction and inflammatory markers according to COVID-19 severity.
