# Supplementary material for: Impact of Coronavirus Disease 2019 on Unresectable Hepatocellular Carcinoma Treated with Atezolizumab/Bevacizumab
Source: J Clin Med. 2024 Feb 27;13(5):1335. doi: 10.3390/jcm13051335 (PMC10931976; doi:10.3390/jcm13051335)
Supplement: Supplementary file 1 [file jcm-13-01335-s001.zip › jcm-2871929-supplementary.pdf]

Supplementary Table S1. Organ dysfunction and inflammatory markers according to COVID severity

No	Age	Sex	ANC (/uL)	Lym (/uL)	NLR	CRP (mg/dL)	LDH (U/L)	COVID severity	*Kidney injury	*Liver injury	*Event
14	59	M	1620.5	756.2	2.1	1.1	218.0	Mild	X	X	X
40	63	M	1015.2	1043.4	1.0	0.7	301.0	Mild	X	X	X
60	51	M	9224.9	2558.2	3.6	11.4	343.0	Moderate	X	O	O
98	41	M	1200.6	930.9	1.3	0.6	339.0	Mild	X	O	O
99	69	F	15580.0	1025.0	15.2	2.8	261.0	Severe	O	O	O
101	46	M	11787.2	1237.1	9.5	19.9	247.0	Moderate	X	O	O
106	44	M	5350.5	2996.7	1.8	0.1	200.0	Mild	X	X	X
108	54	M	761.1	387.0	2.0	0.6	272.0	Asymptomatic	X	X	X
117	61	M	3380.5	931.2	3.6	0.2	197.0	Mild	O	X	X
120	51	M	1617.3	691.2	2.3	0.5	211.0	Mild	X	X	O

124	61	M	1780.9	1680.0	1.1	0.8	220.0	Asymptomatic	X	X	X
126	62	M	3148.7	830.8	3.8	1.2	162.0	Moderate	X	O	O
140	57	M	2253.5	1278.7	1.8	0.2	216.0	Asymptomatic	X	X	X
149	68	M	4320.5	2529.8	1.7	0.4	224.0	Mild	X	X	X
165	62	M	1593.6	172.8	9.2	4.8	401.0	Moderate	O	X	X
170	66	M	1994.3	1178.1	1.7	0.5	258.0	Mild	X	X	X
174	69	M	3554.7	1728.2	2.1	2.3	218.0	Moderate	X	X	X
188	55	M	4229.9	1139.3	3.7	0.8	315.0	Mild	X	X	O
195	53	M	4037.8	2139.0	1.9	0.7	213.0	Mild	X	X	X
196	71	M	6069.5	2239.6	2.7	0.1	203.0	Mild	X	X	X
197	55	F	3205.0	1348.6	2.4	5.4	201.0	Mild	X	X	O
200	62	M	1938.9	1159.2	1.7	0.5	236.0	Mild	X	X	O
211	62	M	13640.6	713.9	19.1	0.6	268.0	Critical	O	O	O
216	65	M	4916.5	768.2	6.4	3.0	120.0	Moderate	O	O	O
217	61	M	1795.5	1050.8	1.7	0.4	317.0	Mild	O	X	X
226	58	F	3581.8	1742.7	2.1	0.1	218.0	Mild	X	X	X

228	57	M	2277.3	770.6	3.0	0.4	224.0	Mild	X	O	O
232	52	M	4992.7	957.6	5.2	7.3	337.0	Mild	X	X	X
235	63	M	3424.9	938.8	3.6	1.4	233.0	Mild	X	O	O
237	71	M	7112.3	1186.9	6.0	0.2	256.0	Asymptomatic	X	O	O
242	60	M	3820.2	908.6	4.2	8.0	432.0	Moderate	X	O	O
243	44	M	3861.2	690.9	5.6	0.1	212.0	Mild	X	X	O
245	71	M	2237.6	581.4	3.8	1.3	191.0	Asymptomatic	X	X	O
248	55	M	2231.0	2898.0	0.8	0.1	164.0	Mild	X	X	X
251	56	M	3474.7	1498.3	2.3	1.9	193.0	Asymptomatic	X	X	O

NLR, neutrophil to lymphocyte ratio; CRP, C-reactive protein; eGFR, estimated glomerular filtration rate

*Event : occurrence of complications or disease progression or death
