# Supplementary material for: Impact of Coronavirus Disease 2019 on Unresectable Hepatocellular Carcinoma Treated with Atezolizumab/Bevacizumab
Source: J Clin Med. 2024 Feb 27;13(5):1335. doi: 10.3390/jcm13051335 (PMC10931976; doi:10.3390/jcm13051335)
Supplement: Supplementary file 1 [file jcm-13-01335-s001.zip › Supplementary figure_.pptx]

## Slide 1
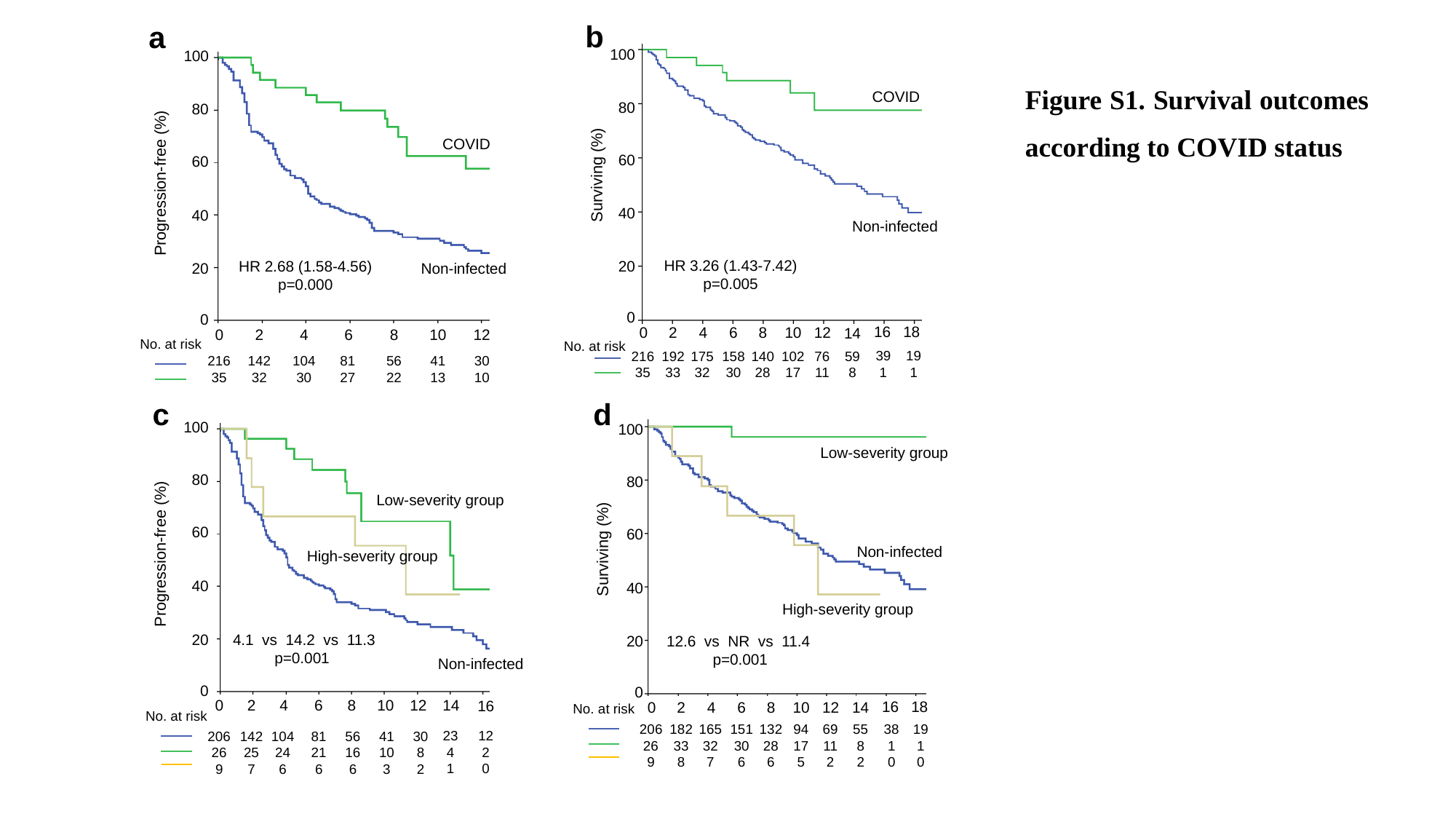

b
a
100
80
60
40
20
0
100
80
60
40
20
0
Figure S1. Survival outcomes
according to COVID status
COVID
COVID
Surviving (%)
Progression-free (%)
Non-infected
HR 3.26 (1.43-7.42)
p=0.005
HR 2.68 (1.58-4.56)
p=0.000
Non-infected
16
18
0
2
4
6
8
10
12
14
0
2
4
6
8
10
12
No. at risk
No. at risk
39
1
19
1
102
17
216
35
19233
175
32
158
30
140
28
76
11
59
8
216
35
142
32
104
30
81
27
56
22
41
13
30
10
d
c
100
80
60
40
20
0
100
80
60
40
20
0
Low-severity group
Low-severity group
Surviving (%)
Non-infected
Progression-free (%)
High-severity group
High-severity group
4.1 vs 14.2 vs 11.3
 p=0.001
12.6 vs NR vs 11.4
 p=0.001
Non-infected
0
2
4
6
8
10
12
14
16
16
18
0
2
4
6
8
10
12
14
No. at risk
No. at risk
38
1
0
19
1
0
94
17
5
206
26
9
18233
8
165
32
7
151
30
6
132
28
6
69
11
2
55
8
2
12
2
0
23
4
1
206
26
9
142
25
7
104
24
6
81
21
6
56
16
6
41
10
3
30
8
2
